# Pyrolysis of Yellow Passion Fruit Peel Waste: A Route to Bio-Oil and Value-Added Chemicals

**DOI:** 10.1021/acsomega.5c10180

**Published:** 2026-08-14

**Authors:** Ana Carolina de Oliveira, Luiz E. P. Borges

**Affiliations:** Catalyst Processes Laboratory, IME-RJ, Praça General Tiburcio N°. 80, Rio de Janeiro, Rio de Janeiro 22290-270, Brazil

## Abstract

This study explores the pyrolysis of yellow passion fruit peel, focusing on bio-oil yields and the chemical characterization of the resulting products under different temperature conditions of biomass drying (373, 378, and 423 K). The liquid product obtained from the pyrolysis process was analyzed using NMR spectroscopy and GC-MS, revealing that acidic groups, ketones, and aromatic compounds predominate in the bio-oil, while alcohols were present in smaller amounts. The effective diffusivity (D_eff_) of moisture during drying was low, suggesting that moisture moves slowly through the peel, which may contribute to the formation of distinct phases in the bio-oil. The obtained diffusivity and activation energy values indicate that moisture transport during drying was diffusion-controlled rather than reaction-controlled. The bio-oil composition analysis showed that high-temperature drying conditions (C) maximized bio-oil yield. The findings suggest that controlled drying of yellow passion fruit peels around 423 K with 1 h of biomass drying optimizes bio-oil production, offering valuable insights for sustainable biomass conversion to bio-oil.

## Graphical abstract

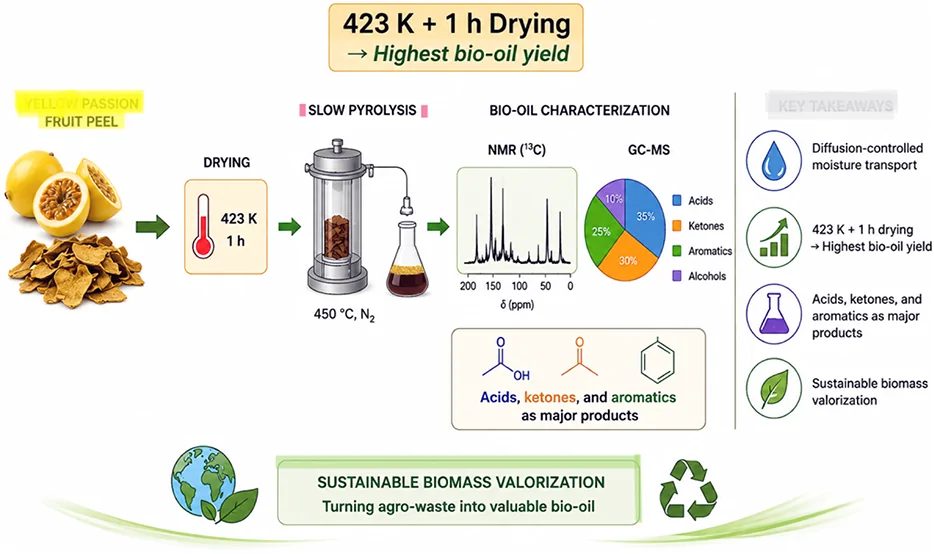

## Introduction

The conversion of lignocellulosic biomass into energy and value-added chemicals is commonly achieved through major thermochemical, biological, and mechanical processes.^1^ Brazil is a major fruit producer, and particularly the world’s largest producer of passion fruit, with a total production of over 600,000 tons and an average yield of 15,259 kg per hectare, according to data from the Brazilian Institute of Geography and Statistics (IBGE, 2021).^2^ The Northeast region stands out, contributing 64.5% of the national production, with the states of Bahia and Ceará among the leading producers. In the Southeast region, the leading producer is the state of Espírito Santo, with 19,347 tons, followed by Minas Gerais with 14,054 tons, making it the fifth-largest producer of passion fruit in the country.^3^ Currently, passion fruit is also cultivated in other tropical climate countries, such as Venezuela, South Africa, Hawaii, and Australia. The spread of passion fruit cultivation promotes the exchange of agricultural techniques and sustainable practices, benefiting producers in different regions with new technologies and knowledge.^4^

Additionally, the presence of passion fruit in international markets increases its visibility and demand, encouraging innovation and continuous improvement in product quality. Finally, passion fruit cultivation in different countries helps preserve biodiversity by promoting the plant’s adaptation to various environmental and climatic conditions, ensuring the sustainability of production in the long term.^4,5^

In many countries, adherence to GLOBAL AP (EuroGAP standards) requires complete traceability across all stages of production and distribution.^5^ While this study was not evaluated under EuroGAP criteria, it strictly adheres to Brazilian National Standards and incorporates the resolutions established by Embrapa, ensuring that traceability and quality control procedures are followed throughout the experimental process.^6^

This biomass has significant potential for reuse in the agro-industrial sector and in thermochemical processes for converting waste materials. The fruit residue is often discarded as wet organic waste, yet it contains hemicellulosic, cellulosic, and lignocellulosic components, as well as pectin and a high moisture content.^7^

The concept of circular reuse, especially of organic matter rich in potential macromolecules, is addressed in the reutilization of organic waste, which is abundant in macromolecules such as cellulose, hemicellulose, and lignin, to create value-added products.^8^ Pyrolysis is one of the techniques that allows the transformation from different feedstocks into bio-oil, char, and other chemicals that can be used in various applications, such as fuels and raw materials for energies industry.^9^ As shown in Table 1.

**Table 1:** Comparative Table of Pyrolysis Yields from Different Feedstocks

Feedstock	Main yield (%)	Ref.
Rice husk, peach pit, sawdust, and other biomasses	Bio-oil: 16–26; Solid residue: 38–48	^10^
Cassava peel	Pyrolytic oil: ∼37.5	^11^
Rice husk	Pyrolytic oil: 32.76; Char: 35.12	^12^
Rice husk	Maximum oil yield ∼56	^13^
Fruit wastes (simulated)	Syngas: 46–55; Biochar: 39–51; Oil <11	^14^
Wood and wood bark	Bio-oil up to 57.7	^15^

A substance is degraded by heat in the absence of an oxidizing agent. During pyrolysis, heat breaks down the original molecular structure of the material. Pyrolysis is widely applied in bioresource management, contributing to energy conversion or the production of value-added chemicals within a circular economy framework.^16^

In Brazil, the large-scale production of tropical fruits generates substantial amounts of peel waste. Pyrolysis represents a viable strategy for reducing this waste while producing valuable compounds that support circular economy goals.^15,16^ The process can be conducted in reactors or microreactor systems, where parameters such as particle size, temperature, heating rate, residence time, inert gas flow, and feedstock properties strongly influence performance.^16^

This study focuses on the characterization of liquid products obtained from slow pyrolysis, providing a detailed assessment of their composition based on biomass drying behavior and kinetic analysis for the obtained diffusivity and activation energy values indicate that moisture transport during drying.

## Experimental Methods

### Sample Preparation

A total of 64 yellow passion fruit samples were randomly purchased from retail markets located in various neighborhoods of a city in the state of Rio de Janeiro, Southeast Brazil. The selection aimed to capture the typical variability found in commercial establishments, without preference for fruit size, supplier, or ripeness. All samples were transported to the laboratory under ambient conditions, washed with distilled water. As shown in Figure 1 the sample was to prepare for analysis, the fruits were first cut in half to allow for a thorough visual inspection of the internal tissues.

**Figure 1:**
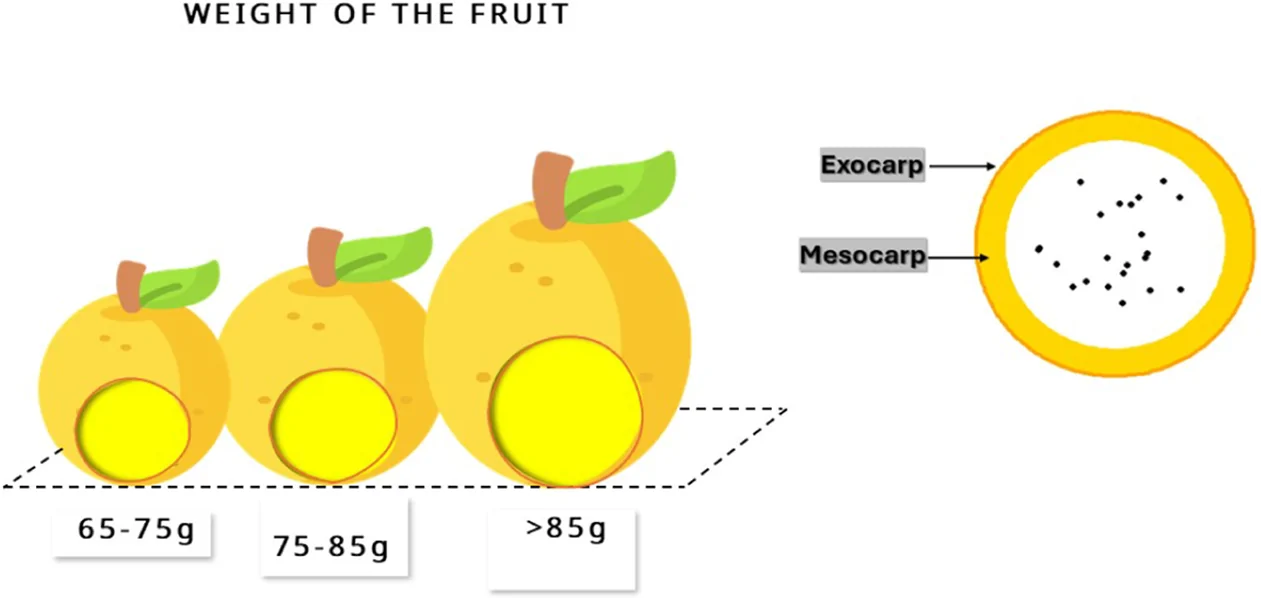
Yellow passion fruit residues used in the mixture follow Brazilian National Standards and Embrapa guidelines.^6^ Image taken by the authors. The image is original, unpublished, and does not contain third-party copyrighted material, trademarks, logos, or identifiable persons.

This procedure ensured that only healthy and uncontaminated material was used. After inspection, the fruit was further sectioned into pieces measuring 0.01 to 0.02 m weighing on the semianalytical balance (MARTE AS5500) Marte Balances and Precision Apparatus Ltd., INMETRO Ordinance DIMEL/108/93 with max. 500g, min load 0.25g. Lowest split 0.01/0.1g, preheat = 20 min. These pieces were then promptly stored at temperatures below 273 K to preserve their integrity and prevent any degradation prior to the pyrolysis tests. The yellow passion fruit used to compose the residue mixture complies with Brazilian National Standards and follows the guidelines established by Embrapa.^6,17^

### Water Content

The water content of the product was determined by gravimetry using an oven at 378 ± 1 K for 24 h, with repetitions. The samples were left at room temperature for 1 h and then placed in a drying oven for 24 h at 378 K, following the methodology proposed in ISSN 15175111 Embrapa Documents 276.^17^

### Moisture Ratios

To determine the hygroscopic equilibrium of yellow passion fruit peel, three replicates of M1.1, M1.2, and M1.3 were prepared, each containing 100 g of sample. The samples were placed in open aluminum trays and dried under identical conditions. Periodic mass measurements were taken until the samples reached a constant weight, in accordance with the stabilization criterion recommended by ISO 18134-3:2015.^18^

The total experimental duration was 1,660 min, with measurements collected at 30 min, 1, 2, 4, 12, and 26 h. The material was arranged in a uniform layer approximately 4 cm (0.04 m) thick to ensure consistent moisture diffusion during drying. The linear regression was performed using MS-Excel (Microsoft 365 MSO Versão 2512 Build 16.0.19530.20184 64 bits).

The moisture ratio of the product was calculated using eq 1:

Equation 1: Moisture ratio of the lignocellulosic biomass.

1 $$\mathrm{MR}=\frac{M_{1}-M_{e}}{M_{0}-M_{e}}$$

where

- *MR* = moisture ratio (dimensionless)
- *M*_*t*_ = moisture content of the sample at time *t* (g water/g dry matter)
- *M*_0_ = initial moisture content of the sample (g water/g dry matter)
*M_e_* = equilibrium moisture content (g water/g dry matter), determined when the mass stabilizes.

Drying Rate Control and Drying of Yellow Passion Fruit Peels

The drying process for yellow passion fruit peels was carried out using a forced circulation oven, specifically the MEMMERT AS5500 LTDA model. For this procedure, the peels were categorized into three types: A, B, and C are summarized Table 2.

**Table 2:** Drying Conditions of the Samples A, B, and C

Samples	Yellow passion fruit peels mass (g)	Temperature (K)
A	30	378 K for a duration of 1 h
B	30	373 K for a duration of 3 h
C	30	423 K for a duration of 1 h

To understand the diffusion process during the drying of yellow passion fruit peel and selected three different drying conditions that could generate varying yields, based on the activation energy from the Arrhenius equation with the linearized curve, as the Newton model was found to be suitable.^19^

Each type underwent distinct drying conditions based on predefined parameters, following the experimental drying kinetics procedure. This approach was designed to better understand the diffusion process, ensuring consistency and facilitating subsequent analysis. The samples were analyzed in replicates. The operating system for samples A, B, and C was the same, according to^20^ and adapted for lignocellulosic biomass as shown Figure 2.

**Figure 2:**
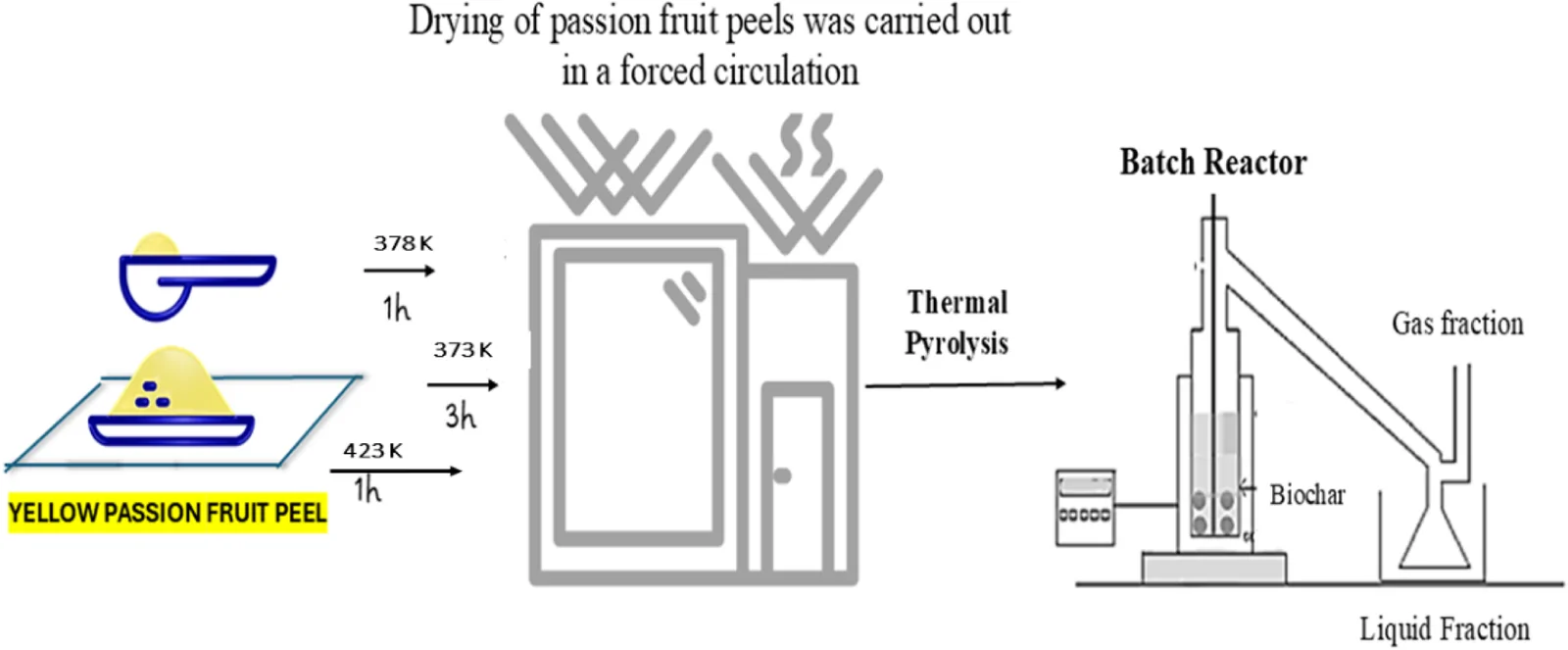
Format of the pyrolysis thermal in a batch reactor.

The yellow passion fruit peels were dried at different temperatures and then weighed. The calculation allows for a balance, aiming at energy efficiency and the reduction of operational costs. Using the Arrhenius equation and models derived from the Fick equation to drying kinetics,^21,22^ and the slope of the line, according with eq 2:

Equation 2: Arrhenius equation and models derived from the Fick equation to drying kinetics.

2 $$\ln\left(D\right)=\ln\left(D_{0}\right)-\frac{E_{a}}{R}\times \frac{1}{T}$$

where:

- *D* is the diffusion coefficient (related to moisture ratio),
- *D*_0_ is the pre-exponential factor,
- *E*_a_ is the activation energy,
- R is the gas constant,
- *T* is the temperature in Kelvin.

### Effective Diffusivity

The effective diffusivity is calculated after determining the activation energy because activation energy is a key parameter used to describe the dependence of diffusivity on temperature. The relationship is exponential, meaning that small changes in temperature or activation energy lead to significant changes in the diffusion rate.^22^ The effective diffusivity (*D*_eff_) was calculated using the following the eq 3:^23^

Equation 3: Equation of effective diffusivity.

3 $$D_{\mathrm{eff}}=\frac{-\mathrm{slope}\times 4L^{2}}{\pi^{2}}$$

### Reactor Heating and Temperature Control

The reactor was heated using a thermal blanket, which provided consistent and uniform heat, ensuring that the temperature was maintained at the desired level throughout the process. The temperature was precisely regulated by a programmable temperature controller, allowing for accurate adjustments based on the specific thermal requirements of the experiment.

This setup ensured that the heating process was reliable and reproducible for all sample runs. The mass of each fraction, divided by the mass of the initial biomass, was used to quantify the yields (bio-oil and biochar). The gas yield was calculated by difference.^24^ According the eq 4:

Equation 4: Yields equation of each fraction products.

4 $$Y\left(\%\right)=\frac{\left(\mathrm{mass}\;\mathrm{of}\;\mathrm{each}\;\mathrm{fraction}\right)}{\left(\mathrm{mass}\;\mathrm{of}\;\mathrm{the}\;\mathrm{initial}\;\mathrm{biomass}\right)}\times 100$$

The difference between samples A, B, and C lies in their drying methods. The operation condition are presented in Table 3.

**Table 3:** Operating Conditions of Pyrolysis for Samples A, B, and C

Sample	Size (m)	Inert Gas	Final Temperature (K)	Residence (min)	Rate (K min^–1^)
A	0.01–0.02	Ar	723	40	5
B	0.01–0.02	Ar	723	40	5
C	0.01–0.02	Ar	723	40	5

The sample, initially the bio-oil has diluted in CH_2_Cl_2_ (Dichloromethane 99% purity, ISOFAR) was vaporized in the heated GC inlet before its vapors sent to the analytical column. The characterization of the gravity-separated aqueous phase remains scarcely explored. In this study, phase separation occurred by gravity. After pyrolysis, the samples were stored at 277 K for 15 days to ensure complete phase settling, following an established methodology previously adapted for rubber pyrolysis experiments.^24^

GC-MS analysis was performed on the Shimadzu equipment, model Nexis GC–2030, coupled to a mass spectrometer, with an RTX 5MS silica capillary column. The autosampler injects the sample into the GC inlet, introducing it into the carrier gas He, summarized in Table 4. The samples A, B, C organic phase and aqueous phase was plotted using RStudio (Windows NT 10.0; Win64; 2009–2026 Posit Software, PBC).

**Table 4:** Operating Conditions GC-MS Analysis

	Injector and interface T (K)		Flow (mL. min^-1^)	Split
Injector	553		1.0	1:100
**Oven**		Ratio (K. min)	Final temperature (K)	Hold (min)
		10	523	2
		15	548	8
		20	573	10
**MS**		**Initial (*m*/*z*)**	**Final (*m*/*z*)**	
		50 u	500 u	

The silica capillary column measures 30 m × 0.25 mm × 0.25 mm, with helium as the carrier gas. The mass spectrometer operated with 70 eV electron impact ionization. The database used was NIST (National Institute of Standards Reference and Technology).

Nuclear magnetic resonance (NMR) spectra of ^13^C and ^1^H were recorded using a Varian PREMIUM COMPC spectrometer operating at 600 MHz. CDCl_3_ was used as the solvent, and tetramethylsilane (TMS) was employed as an external standard for spectra acquired in D_2_O.

## Results and Discussion

Determining the activation energy is essential for understanding the drying kinetics of yellow passion fruit peels, which water contain 83% of which water was applied as a pretreatment for passion fruit peels before separating them into three fractions (A, B, C). As shown in Table 5 the model used to relate moisture ratio and temperature over time for this evaluation.

**Table 5:** Model Adjustment for Determination of Activation Energy of Yellow Passion Peels

Moisture Ratio	ln *M*R	T (min)
0.995301	–0.00471	90
0.968072	–0.03245	150
0.804008	–0.21815	330
0.750795	–0.28662	400

The Newton drying model is a purely empirical expression that assumes first-order drying kinetics, in which the moisture removal rate is proportional to the remaining moisture content. Although it provides a simple description of the drying behavior, it does not explicitly account for mass transfer mechanisms such as internal diffusion, external convection, or structural effects.^23–25^ The inclusion of this model in Figure 3 serves as reference and for identification the general trend of the experimental data.

**Figure 3:**
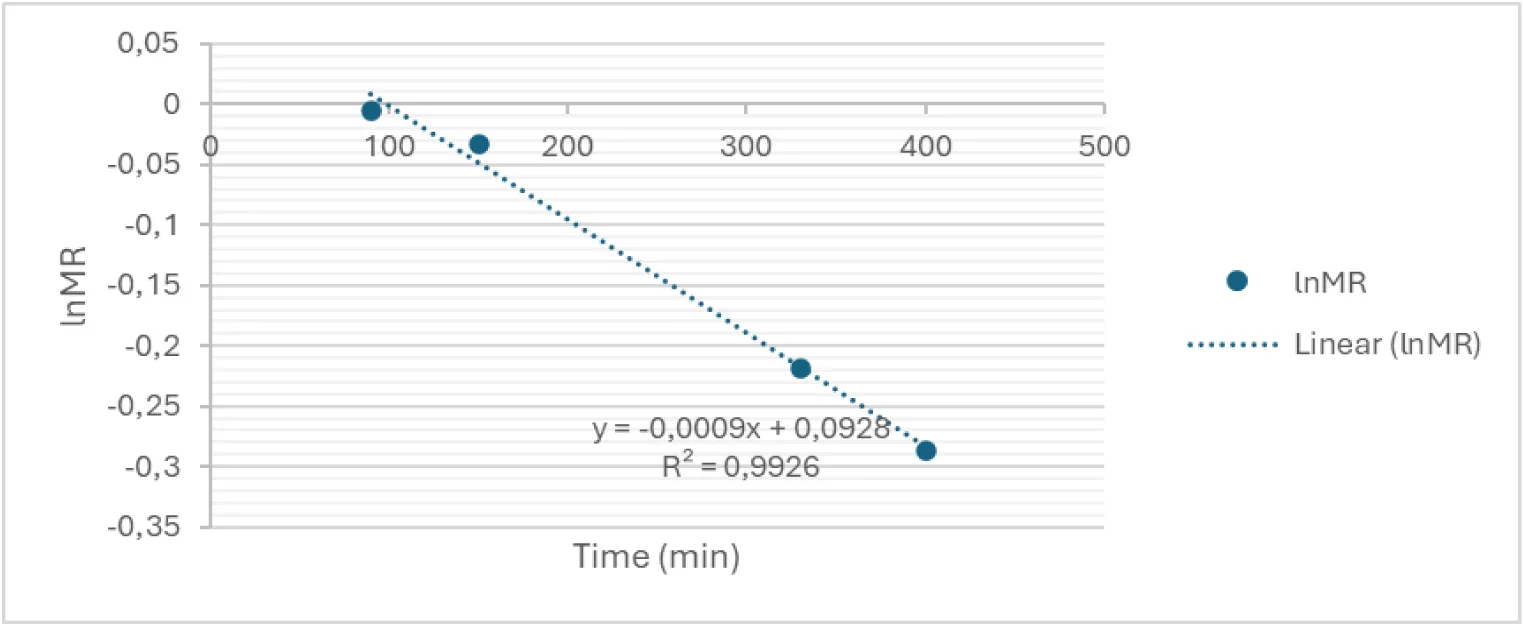
Newton model with the linearized curve.

The curve as shown in Figure 3, Slope: −0.0009 min^–1^, Intercept: 0.0928 (ln units), R^2^ (ln fit): 0.9926. Given the temperature of 378 K. The activation energy for the drying process at 1 h and is approximately:

$$E_{a}\approx 0.00748\;J\;mol^{-1}$$

Given the temperature of 373 K and the drying time of 3 h, the calculated activation energy is approximately:

$$E_{a}\approx 0.00748\;J\;mol^{-1}$$

And drying process at 1 h and 423 K is approximately:

$$E_{a}\approx 0.00748\;J\;mol^{-1}$$

The biomass analysis of residue samples A, B, and C included the determination of activation energy associated with their drying behavior. This evaluation was performed by relating moisture ratio and temperature over time, allowing the characterization of the drying kinetics for each sample.^26^ The experimental results and the fitted model parameters used to obtain the activation energy values are summarized in Table 6.

**Table 6:** Biomass Analysis of Residue Samples A, B, and C with the Activation Energy for the Drying Process

Samples	Yellow passion fruit peels mass (g)	Temperature	Activation energy (J mol ^–1^)
A	30	378 K for a duration of one hour	0.00748
B	30	373 °C for a duration of three hours	0.00748
C	30	423 K for a duration of one hour	0.00748

The activation energy values obtained from the Newton drying model showed minimal sensitivity to variations in temperature and drying time, consistent with the empirical nature and known limitations of this model,^27^ which does not explicitly represent mass transfer mechanisms, including internal diffusion, external convection, or structural effects. Accordingly, the resulting activation energy should be interpreted as an apparent kinetic parameter.

However, the approximately linear behavior observed in the drying curves enabled the estimation of the slope, which justified the use of Fick’s diffusion model as an auxiliary framework for the analysis of moisture transport.^28^ The effective moisture diffusivity under different drying conditions was estimated by plotting ln­(MR) versus drying time and using the slope method. The slope of the regression equation was calculated by using the experimental data within the period when the drying rate decreased.^29^

The slope of the line (0.0009) is effectively representing the overall diffusion process, which can be used to estimate the energy required for the process. The effective diffusivity (*D*_eff_) was calculated using the following equation:^29^

$$D_{\mathrm{eff}}=D_{0}\;\exp\left(-E_{a}|RT\right)$$

Using the slope value from the experimental data (−0.0009) and the characteristic peel length (L = 0.04 m) from the previous experimental methods, we can now substitute these values into the formula:

$$(D_{\mathrm{eff}})=5.83\times 10^{-7}\;m^{2}\;s^{-1}$$

This value represents the rate at which moisture diffuses through the yellow passion fruit peel during the drying process. This is a small value, as expected in drying processes, meaning that moisture moves relatively slowly through the material. The effective diffusion in studies others agricultural products, was reported in Table 7.

**Table 7:** Effective Diffusion Coefficients for Selected Agricultural Materials

Material	*D*_eff_ (m^2^ s^–1^)	Source
Passion fruit peel (this work)	5.83 × 10^–7^	-
Banana (conv.)	1.57–2.63 × 10^–10^	^30^
Banana (green peel)	5–7 × 10^–8^	^31^
Mango foam-mat	0.81–5.75 × 10^–9^	^32^
Mango slices	1.74–3.15 × 10^–10^	^33^
Orange-like (citrus peels)	∼7.89 × 10^–10^–1.6 × 10^–9^	^34^

The effective moisture diffusivity obtained for passion fruit peel (5.83 × 10^–7^ m^2^ s^–1^) falls within the broad range reported for fruits and vegetal materials (10^–11^–10^–9^ m^2^ s^–1^). Specifically, values for banana slices have been reported between ∼1.6 × 10^–10^ and 8.0 × 10^–9^ m^2^ s^–1^ at similar drying conditions, consistent with our results. Orange peel studies report lower diffusivity ∼7.89 × 10^–10^–1.6 × 10^–9^ m^2^ s^–1^, possibly due to structural and compositional differences. Meanwhile, in mango foam-mat drying, higher diffusivity values (0.8 × 10^–9^ to 5.8 × 10^–9^ m^2^ s^–1^) have been observed, likely influenced by sample preparation and drying methods.

This phenomenon indicated a greater magnitude of water transport from the interior to the exterior of the product, corroborating with on drying kinetics and effective diffusivity in fruits peels.^30,31^ A process similar to that found pointed out that the effective diffusion coefficients are in the range of 10^–10^ to 10^–9^ m^2^ s^–1^ for drying.^32–34^ The fact that moisture moves slowly and is not completely removed all at once may result in different thermal conditions in parts of the material during pyrolysis, leading to the formation of distinct phases in the bio-oil.^35^ Also indicate that different types of compounds are being generated at different stages of the process, resulting in a separation into two phases. Bio-oil can be used as fuel directly, according to ASTMD7544 or can be upgraded to high-grade fuels or chemical materials.^35,36^ In addition, the volatile organic composition of solvent CHCl_3_ was analyzed for GC-MS fraction liquid of pyrolysis (A, B, C samples). The results are summarized in Table 8. The blank provides a baseline profile of the system, allowing the identification of background signals originating from the instrument, column bleed, solvents, or potential contaminants introduced during sample preparation and handling.

**Table 8:** Identification of Volatile Organic Compounds of Solvent CHCl_3_ Was Obtained Using GC-MS

Area (%)	*m*/*z*	Name
14.86	91.10	Toluene
77.24	165.90	Tetrachloroethylene
3.54	91.10	Benzene, 1,3-dimethyl
1.46	149.05	Bis(2-ethylhexyl) phthalate

As shown in Figure 4, the yield of pyrolysis process of yellow passion fruit peel in three different conditions of pretreatment (solid fraction, liquid fraction, and gaseous fraction).

**Figure 4:**
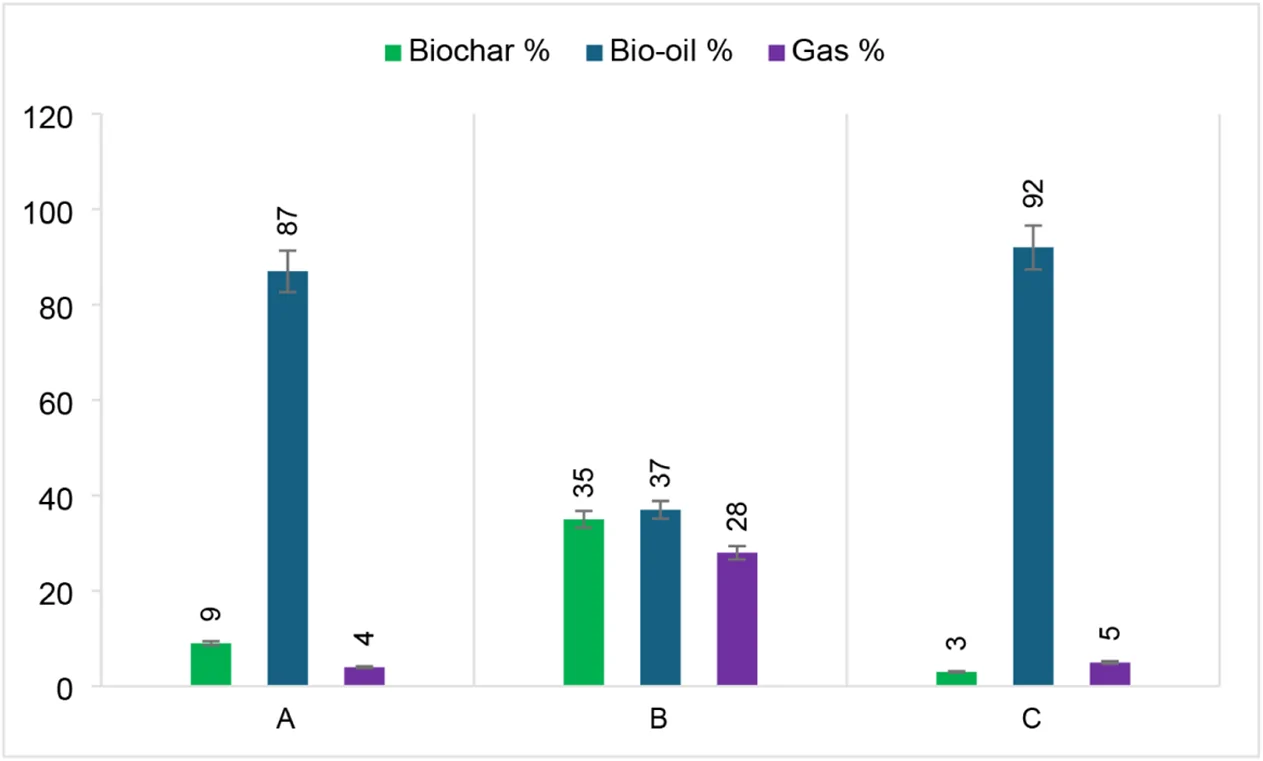
Experimental yields from thermal pyrolysis of yellow passion fruit peel.

### Sample A

Sample A shows a high percentage of bio-oil. This indicates that, under the conditions of 378 K for a duration of 1 h, the production of gas or biochar is not more predominant. These conditions may be associated with a process that favors the decomposition of biomass into liquid conversion.

### Sample B

In sample B, bio-oil appears in a less amount than in A, and there is still a significant proportion of biochar. This suggests an intermediate process or condition, at 373 K for a duration of 3 h, where the balance between the three products is more even. The pyrolysis process may be adjusted to generate reasonable amounts of bio-oil, but biochar formation has not been neglected, possibly due to a lower temperature or a conversion rate that favors both liquids and solids.

### Sample C

Sample C stands out, with the highest percentage of bio-oil relative to the other products. This may indicate that the pyrolysis conditions and operational parameters at 423 K for a duration of 1 h favored the conversion of biomass into liquids, minimizing the production of gas and biochar. For these conditions, pyrolysis maximized bio-oil production, a liquid fraction with higher commercial value in various applications, such as fuel and chemicals.

Additionally, sample C may have been inadvertently exposed to a basic solution during handling. Although this hypothesis was not experimentally confirmed, the presence of a basic solution in close proximity to the sample is reported here for transparency. This represents a potential experimental limitation that may have influenced the observed drying behavior.

It is important to note that drying conditions play a critical role in mass transfer phenomena. Short-time, high-temperature drying processes differ significantly from prolonged drying at temperatures below 100 °C, as reflected in the adopted linearized model. Accordingly, the observed deviations are discussed considering both potential external influences and the intrinsic sensitivity of the drying process.

Figure 5 illustrates the percentage area of various chemical functional groups of the A, B, and C samples for the respective organic and aqueous phases. The GC–MS results are presented by grouping the identified compounds according to their functional classes, with their relative abundances expressed as the sum of the corresponding peak areas. This approach allows a more systematic interpretation of the data by emphasizing the distribution of chemical functionalities rather than individual compounds.

**Figure 5:**
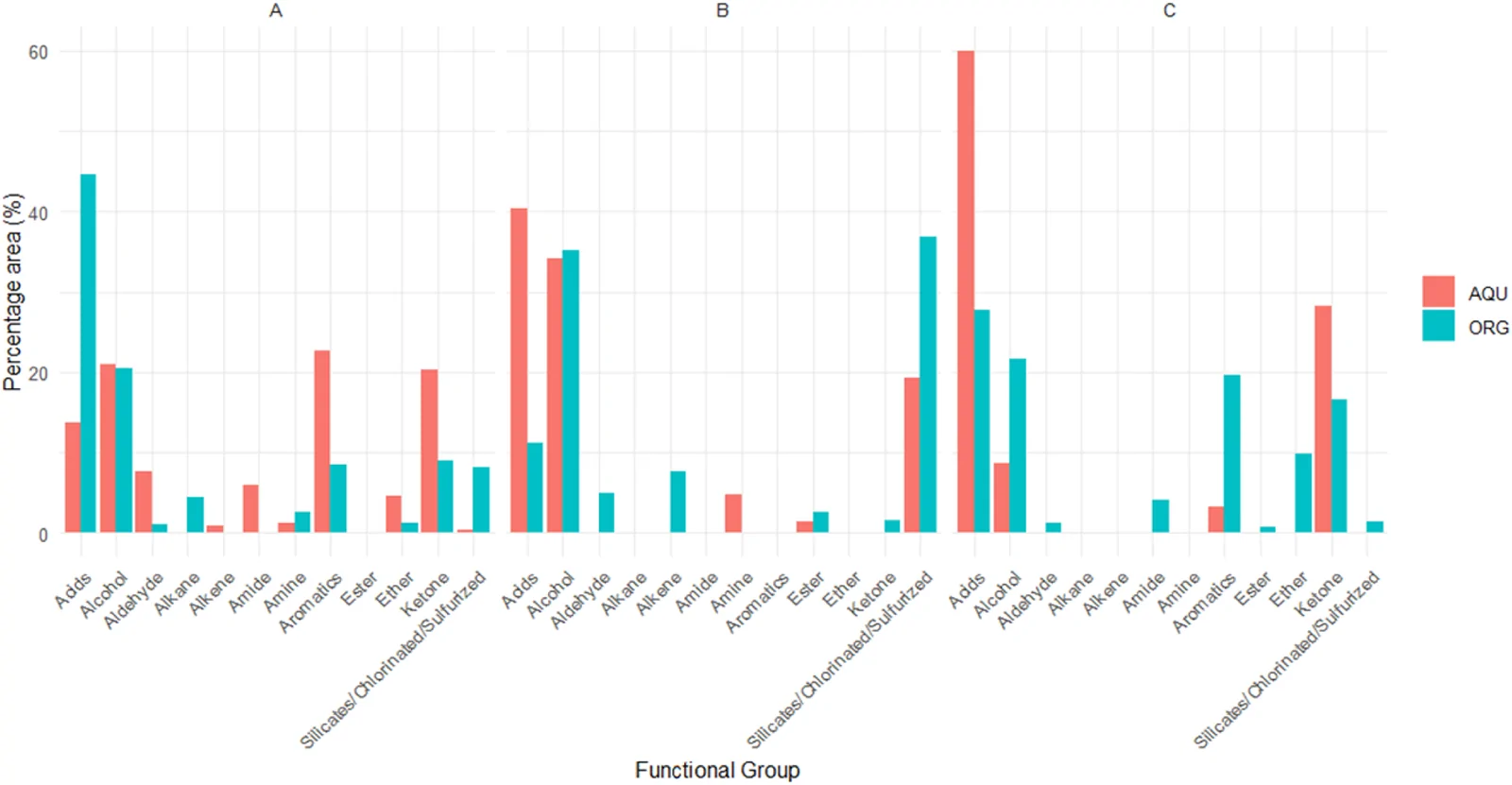
Organic and aqueous phases of samples A, B, and C.

Bio-oil contains oxygenated, nonoxygenated, and nitrogen-containing compounds. In slow pyrolysis, the bio-oil separates into two liquid phases: an organic phase and an aqueous phase. Composition by GC-MS of the organic and aqueous phases of samples A and B (Appendix).

### Sample A

Acids are the most significant group here. Interestingly, they are much higher in the ORG phase (∼45%) compared to the AQU phase (∼15%). Secondary groups: Aromatics and ketones show the opposite trend to acids, with significantly higher percentages in the AQU phase than the ORG phase. Alcohols are roughly balanced between the two phases (around 20%).

### Sample B

Acids and alcohols dominate, but the distribution shifts. Acids: The trend flips compared to sample A. Here, acids are much higher in AQU (∼40%) than in ORG (<15%). Silicates/Chlorinated/Sulfurized: This group shows a massive spike, particularly in the ORG phase (∼38%) and the AQU phase (∼19%). Minor Groups: Alkenes appear in the ORG phase but are absent in the AQU phase.^37^

### Sample C

Acids show the highest peak of the entire chart in the AQU phase (hitting ∼60%), while the ORG phase is around 28%. Secondary Groups: Ethers become significant here, with a higher presence in AQU (∼29%) than ORG (∼18%). Aromatics: Unlike sample A, the aromatics here are much higher in the ORG phase (∼20%) compared to the AQU phase (<5%).^38^

Solubility depends on attractive intermolecular forces short-chain fatty acids (SCFAs), also called volatile fatty acids, are organic linear carboxylic acids with fewer than six carbons, including acetic acid, propionic acid, butyric acid, and valeric acid.^39^

Acids represent the most volatile group among the compounds analyzed. In sample A, acids are predominant in the organic (ORG) phase. However, this trend shifts in samples B and C, where acids become the dominant functional group in the aqueous (AQU) phase. Alcohols are consistently present across all three. Their distribution remains relatively balanced between the organic and aqueous phases compared to other functional groups, indicating a steady solubility profile regardless of the experimental condition. The abundance of ketones, aromatic compounds, and alcohols is directly associated with the intrinsic composition of the biomass, particularly its high lignin content. As a highly aromatic biopolymer composed of phenylpropanoid units, lignin, upon thermal degradation, predominantly promotes the formation of phenolic and aromatic derivatives, as well as ketonic compounds generated through secondary cracking and rearrangement reactions.^40,41^

In comparison, alcohols are mainly derived from carbohydrate fractions, such as cellulose and hemicellulose, which give rise to more oxygenated and thermally less stable intermediates. Under the applied thermal conditions, these intermediates tend to undergo subsequent dehydration, decarboxylation, and cracking reactions, leading to the formation of ketones, aldehydes, and aromatic compounds.^35,40^

The primary reaction pyrolysis occurs when the initial products are produced from biomass heating. The result of the sample’s dehydration, followed by the release of volatile compounds and a longer time in the reactor, causes secondary and tertiary reactions. The sample in pyrolysis process with can involve a process of removal or conversion of oxygenated compounds into carboxyl, which is formed by a carbon atom linked to a carbonyl group (C=O) and a hydroxyl group (OH), modifying their properties.^40,41^

The NMR spectra of the bio-oil organic phase from samples A and B were acquired using CDCl_3_ (CAS: 865-49-6) as solvent.

Due to experimental limitations related to sample availability and solvent consumption, it was not possible to reacquire additional high-quality ^1^H NMR spectra for samples B and C suitable for publication. For sample C, which exhibited the highest bio-oil yield, ^13^C NMR spectra of the aqueous phase were acquired using D_2_O as solvent, with tetramethylsilane (TMS) as the reference standard.^42^

The ^13^C DEPT (Distortionless Enhancement by Polarization Transfer) NMR spectrum is a powerful tool for investigating intra- and intermolecular dynamic processes. The obtained ^1^H and ^13^C NMR spectra exhibited very similar spectral profiles; therefore, to avoid redundancy, only the ^13^C DEPT spectrum of sample A. Figure 6 presents a detailed structure of the organic bio-oil sample. The signals are well resolved, with no significant overlap. In particular, it allows the identification of intramolecular (fluxional) processes such as donor group migration, bond rotation, conformational changes, and ring flipping. In the present spectrum, the signals are well resolved and show no significant overlap.

**Figure 6:**
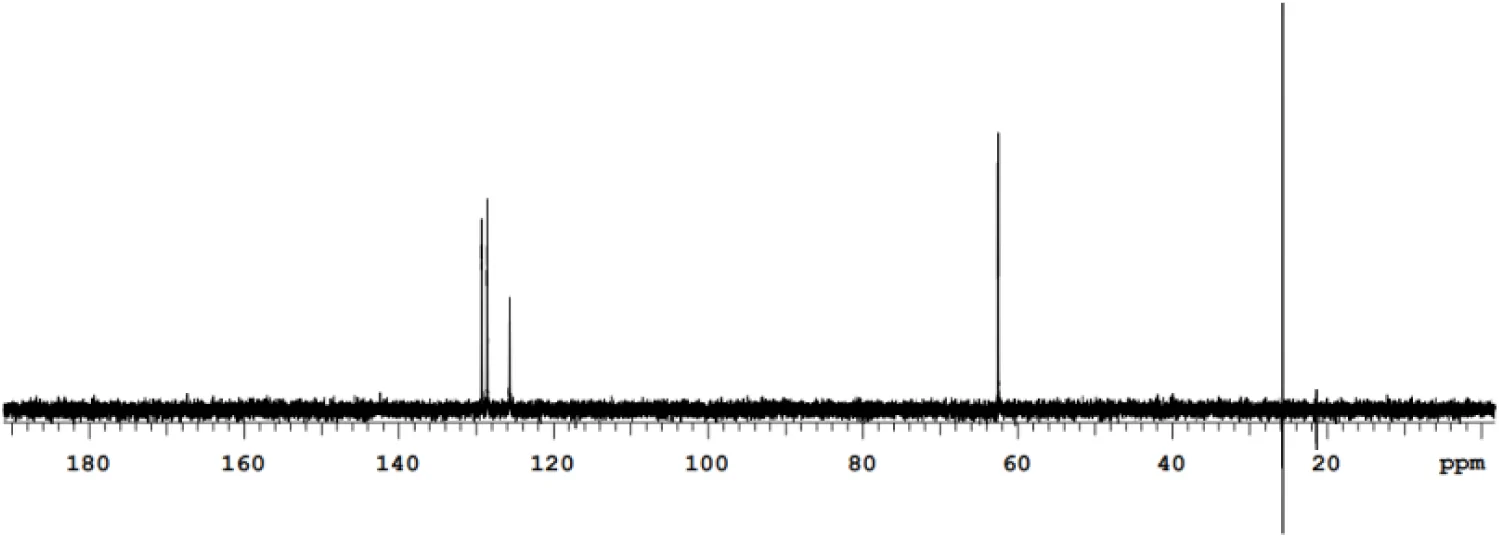
^13^C NMR DEPT spectrum of the organic phase of sample A.

The DEPT-135 spectrum confirmed the presence of CH and CH_3_ carbons through positive resonances, while CH_2_ carbons appeared as negative signals. Quaternary carbons were not detected in the DEPT-135 experiment due to the absence of directly bonded hydrogens. The DEPT-135 spectrum showed positive resonances around 25 ppm, attributed to aliphatic CH_3_ and/or CH groups, while negative signals in the same region were assigned to CH_2_ carbons.^42,43^ Additionally, positive signals observed between 120 and 140 ppm indicate the presence of protonated olefinic and/or aromatic carbons.^43^ These results confirm that the bio-oil contains both aliphatic hydrocarbon chains and unsaturated structures, consistent with the compounds identified by GC–MS analysis.^42–44^

The signs (*UP*) are saturated with methyl carbons (−CH_3_). The signs (*DOWN*) are assigned to methylene carbons (−CH_2_).^42^ In addition to observing that the shortest and longest signals from carbon are related to the relaxation time that these protons have.^42^ According to the spectroscopic analysis of the bio-oil, sample A shows well-defined carbon skeletons. NMR ^1^H sample A organic phase is cleary visible in Figure 7.

**Figure 7:**
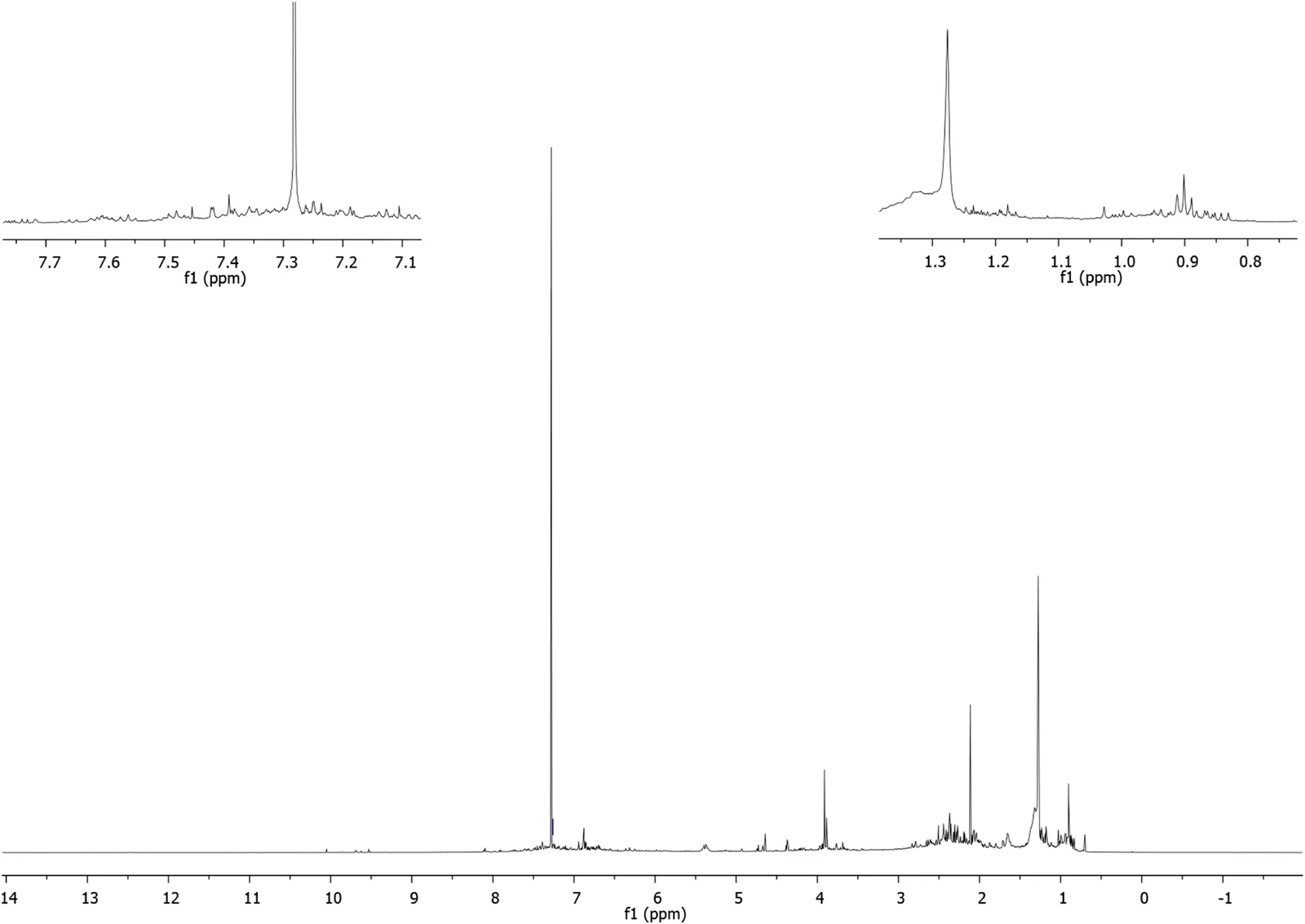
^1^H NMR spectrum of the organic phase of sample A.

The spectrum indicates the presence of hydrogen in the low-field and high-field regions. The chemical displacement of aromatic hydrogens occurs in the most unprotected region. And the presence of electronegative atoms near the hydrogens is noted (ROH, HON, OCH_3_) from δ 4.00 to 1.00 ppm.^42^ The signals between 4 and 5 ppm in the ^1^H NMR spectrum correspond to hydrogens attached to alcohol carbons. These signals are typically observed for hydroxyl groups (−OH) or alcohol in organic compounds. This range is characteristic because the alcohol proton is typically deshielded due to its ability to form hydrogen bonds, which affects the chemical shift of the proton.

In sample B, an interesting feature is the signal near ∼10 ppm, which corresponds to the acid hydrogen in a carboxyl group (R–CO_2_H). This proton is typically highly deshielded due to the electron-withdrawing effect of the carbonyl group (C=O), resulting in a downfield shift (to the higher ppm region). The sharpness and intensity of the signal at this position reflect the presence of a carboxylic acid group (−CO_2_H) in the structure.^43^

The loss of this proton, as suggested in the result, is favored because of the stabilization of the conjugate base (RCOO^–^). This occurs through electronic delocalization of the negative charge over the carboxylate anion. The negative charge on the oxygen atom in the conjugate base is stabilized by resonance, allowing the proton to dissociate more readily. This phenomenon is typical of carboxylic acids, where the proton dissociation is driven by the ability of the conjugate base to stabilize the resulting negative charge.^43^

Therefore, the presence of the carboxylic acid hydrogen signal at ∼10.00 ppm in sample B indicates that the compound exists in an equilibrium, where the proton may dissociate, especially under conditions that favor the stabilization of the conjugate base.^42,43^ This is an important observation in understanding the acidic behavior of the sample and how its structure can contribute to the overall chemical reactivity, especially in processes such as pyrolysis or other chemical reactions where proton loss may occur.

Almost all NMR solvents contain residual amounts of water. In addition, many commonly used solvents are hygroscopic and readily absorb moisture from the atmosphere, leading to an increase in water content over storage time. The presence of a water-derived signal (HDO) can significantly degrade the quality of NMR spectra, reducing spectral resolution, particularly in aqueous systems.^43^ According to the spectroscopic analysis of the bio-oil, sample C shows a higher yield for the aqueous phase in its NMR ^13^C spectrum. Figure 8 illustrates the overral spectra of the aqueous phase.

**Figure 8:**
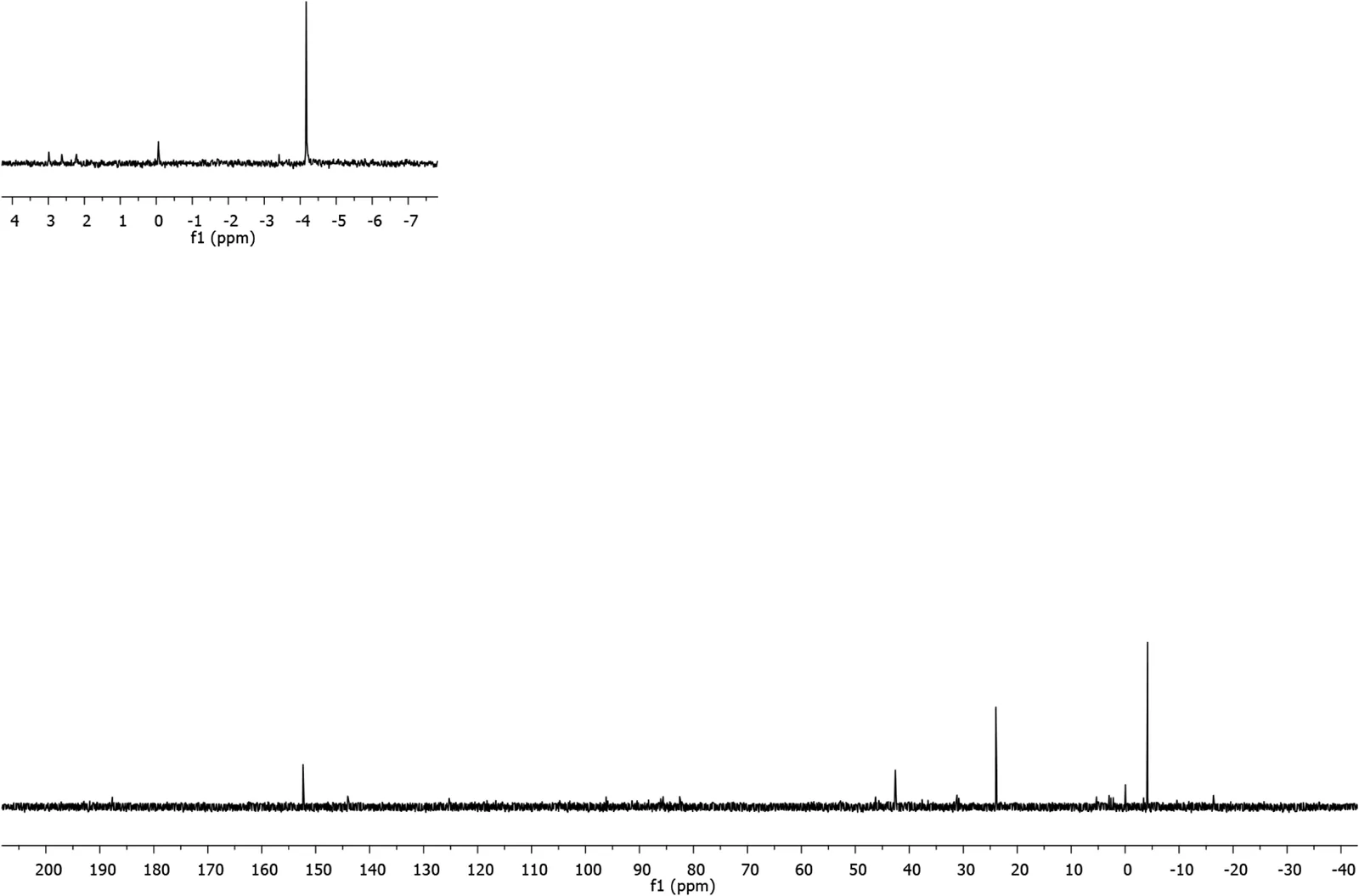
^13^C NMR spectrum of the aqueous phase of sample C.

In the ^13^C NMR spectrum of the aqueous phase of the bio-oil, the resonance at approximately 35 ppm assigned to CH_2_ groups from aliphatic chains, indicating the presence of low-molecular-weight aliphatic fragments derived from the thermal cleavage of polymeric constituents of the biomass. These signals are consistent with secondary cracking reactions occurring during pyrolysis, which generate short-chain aliphatic structures that remain soluble in the aqueous fraction.^42,43^

Additionally, a single at around alkenes and aromatic carbon atom (100–170 ppm) is attributed to 43 ppm (−CH_2_−) carbon located between two carbonyl groups, characteristic of α-carbon environments in diketone structures.^42,43^ The presence of such diketone-type compounds suggests the formation of oxygenated species through dehydration, fragmentation, and rearrangement reactions of carbohydrate-derived intermediates during pyrolysis. Together, these resonances highlight the predominance of oxygen-rich aliphatic compounds in the aqueous bio-oil fraction and reflect the multiple reaction pathways involved in biomass thermal decomposition.^44^

## Conclusions

The primary chemical differences among samples A, B, and C attributed to biomass residue drying. The initial moisture content of 83% indicates a high-water concentration in the residual solid. This study confirmed that the two phases of liquid product result from slow pyrolysis. In addition to presenting the highest bio-oil yield, the pretreated sample C (423 K for 1 h) also contains a greater amount of volatile matter compared to the untreated sample. The different samples, as sample C may have been inadvertently exposed to a basic solution during handling. Although not experimentally confirmed, a basic solution was present near the sample, and this possibility is acknowledged for transparency.

This represents an inherent experimental limitation and may have influenced the observed drying behavior of sample C. It is important to note that drying conditions strongly affect mass transfer: short-time/high-temperature drying differs significantly from prolonged drying below 100 °C, as reflected in the linearized model. Therefore, the observed deviations are interpreted considering both potential external influences and the intrinsic sensitivity of the drying process.

As reported in the literature, the low drying rate of lignocellulosic biomass reflects the difficulty of removing bound water from the solid during the drying process:

1. A favors the extraction of acids into the organic phase.
2. B and C favor the retention of acids in the aqueous phase.
3. This observation is consistent with the formation of a biphasic pyrolysis liquid (aqueous and organic phases) flow properties for the process drying.

Furthermore, the ^13^C and ^1^H NMR spectra of samples A, B, and C exhibited very similar patterns, suggesting that the experimental conditions similarly influenced the chemical structures in these samples, with a consistent distribution of these compounds such as methyl groups (CH_3_) or methylene (CH_2_) in aliphatic and (H–O) chains of alcohols. The ketones and aromatics present in higher amount than alcohols.

The higher abundance of ketones and aromatic compounds compared to alcohols can be attributed to the intrinsic composition of the biomass, particularly its lignin content. Lignin is a highly aromatic biopolymer composed of phenylpropanoid units, and its thermal degradation predominantly generates phenolic and aromatic derivatives, as well as ketonic structures formed through secondary cracking and rearrangement reactions.

In contrast, alcohols are mainly derived from carbohydrate fractions such as cellulose and hemicellulose, which tend to produce more oxygenated and less stable intermediates. These compounds are more susceptible to further dehydration, decarboxylation, and cracking reactions under the applied thermal conditions, leading to the formation of ketones, aldehydes, and aromatics.

Therefore, the predominance of ketones and aromatics reflects both the relatively higher lignin contribution in the biomass and the thermal instability of alcohol intermediates during the process.

## Figures and Tables

**Figure 9:**
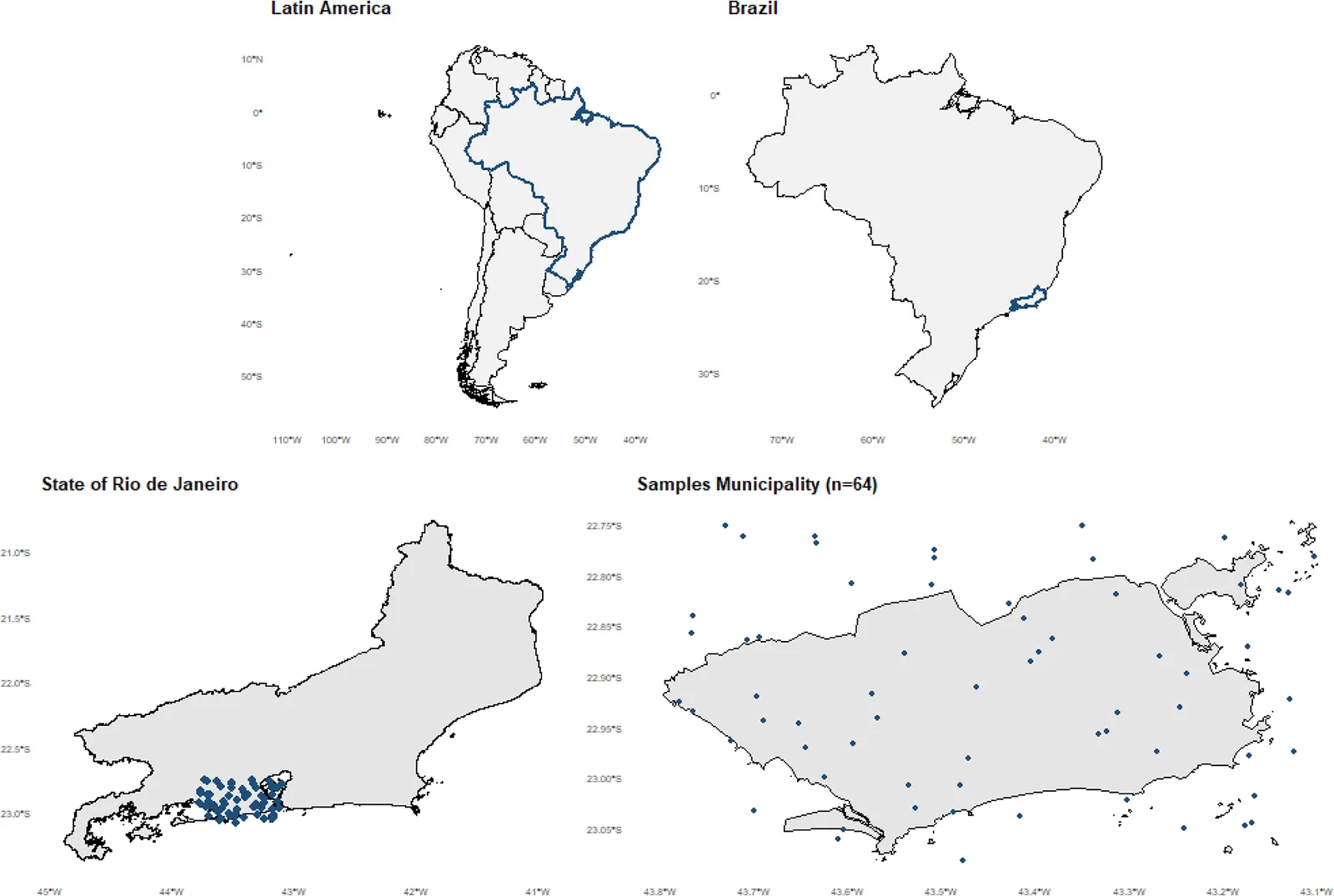
Locations where samples were acquired within the study area, bounded by coordinates 22°54′10″–22°54′23″ S and 43°10′21″–43°12′28″ W. Map created by the authors using posit team (2025). RStudio: integrated development environment for R. Posit software, PBC, Boston, MA. http://www.posit.co/. Geospatial data plotting and base map retrieval were performed in the R environment using the ggmap package.^45^

## Appendix

### Model Adjustment for Determination of Activation Energy

To determine the activation energy, an appropriate model adjustment is crucial for accurately describing the drying process. Typically, the Arrhenius equation is used to relate the diffusion rate to temperature, as it accounts for the temperature dependence of the process.^31^ This equation is often linearized, allowing the activation energy to be determined from the slope of the plot. The general form of the Arrhenius eq 5:

Equation 5: The activation energy with appropriate model adjustment.

5
$$D=D_{0}\times e^{-\frac{E_{a}}{RT}}$$

where:

- *D* is the diffusion coefficient (or drying rate),
- *D*_0_ is the pre-exponential factor,
- *E*_a_ is the activation energy,
- *R* is the gas constant,
- *T* is the absolute temperature in Kelvin.

After linearizing the equation, it is becomes eq 6:

Equation 6. The activation energy with linearizing appropriate model adjustment

6
$$\ln(D)=\ln(D_{0})-\frac{E_{a}}{R}\times \frac{1}{T}$$

By plotting ln­(*D*) against 1/*T*, the activation energy (*E*_*a*_) can be calculated from the slope of the resulting line eq 7:

Equation 7. Equation of activation energy based on data experimental.

7
$$E_{a}=-\mathrm{slope}\times R$$

This approach allows for the determination of activation energy based on experimental, and the accuracy of the linear model can be validated using statistical methods such as R^2^ ensuring a reliable estimation of the kinetic parameters. Figure 9 presents a detailed localization of sampling groups fruits.
